# Risk of Liver Injury Associated with Intravenous Lipid Emulsions: A Prescription Sequence Symmetry Analysis

**DOI:** 10.3389/fphar.2021.589091

**Published:** 2021-03-01

**Authors:** Xiao-xiao Li, Yin-chu Cheng, Suo-di Zhai, Peng Yao, Si-yan Zhan, Lu-wen Shi

**Affiliations:** ^1^ Department of Pharmacy, Peking University Third Hospital, Beijing, China; ^2^ Department of Pharmacy Administration and Clinical Pharmacy, School of Pharmaceutical Sciences, Peking University, Beijing, China; ^3^ Department of Epidemiology and Biostatistics, School of Public Health, Peking University Health Science Center, Beijing, China; ^4^ Research Center of Clinical Epidemiology, Peking University Third Hospital, Beijing, China

**Keywords:** lipid emulsion, hepatic protector, prescription sequence symmetry analysis, pharmacoepidemiology, health insurance database, drug safety

## Abstract

**Aims:** To determine the risk of liver injury associated with the use of different intravenous lipid emulsions (LEs) in large populations in a real-world setting in China.

**Methods:** A prescription sequence symmetry analysis was performed using data from 2015 Chinese Basic Health Insurance for Urban Employees. Patients newly prescribed both intravenous LEs and hepatic protectors within time windows of 7, 14, 28, 42, and 60 days of each other were included. The washout period was set to one month according to the waiting-time distribution. After adjusting prescribing time trends, we quantify the deviation from symmetry of patients initiating LEs first and those initiating hepatic protectors first, by calculating adjusted sequence ratios (ASRs) and relevant 95% confidence intervals. Analyses were further stratified by age, gender, and different generations of LEs developed.

**Results:** In total, 416, 997, 1,697, 2,072, and 2,342 patients filled their first prescriptions with both drugs within 7, 14, 28, 42, and 60 days, respectively. Significantly increased risks of liver injury were found across all time windows, and the strongest effect was observed in the first 2 weeks [ASR 6.97 (5.77–8.42) ∼ 7.87 (6.04–10.61)] in overall patients. In subgroup analyses, female gender, age more than 60 years, and soybean oil-based and alternative-LEs showed higher ASRs in almost all time windows. Specially, a lower risk for liver injury was observed in the first 14 days following FO-LEs administration (ASR, 3.42; 95% CI, 0.81–14.47), but the risk started to rise in longer time windows.

**Conclusion:** A strong association was found between LEs use and liver injury through prescription sequence symmetry analysis in a real-world setting, which aligns with trial evidence and clinical experience. Differences revealed in the risks of liver injury among various LEs need further evaluation.

## Highlights

•Drug safety signal was detected by using prescription sequence symmetry analysis and Chinese Basic Health Insurance database.
•Lipid emulsion use in the first 2 months is associated with 3.6- to 7.9-fold increased risk for liver injury requiring use of hepatic protectors in a real-world setting.
•The association is positive across all time windows and generations.
•It suggests that liver injury after lipid emulsion initiation is common in China.
•It is important to strengthen the appropriate use of LEs at the beginning of administration.

## Introduction

Patients dependent on total or partial parenteral nutrition (PN) are at higher risk of developing a wide range of disruption to liver function, such as cholestasis and steatosis (Beath and Kelly, 2016; Meyerson and Naini, 2019). It occurs as a major consequence of metabolic complications related to PN, besides physiological or anatomical abnormalities, with an incidence ranging from 20% to 80% in both adults and children (Gabe, 2013).

As an integral component of PN, a wide variety of commercial lipid emulsions (LEs) is now available. However, the amount, type, and infusion time of intravenous LEs were reported to affect the risk of inducing liver complications (Badia-Tahull et al., 2015; Kapoor et al., 2019a; b). Exposure to excess intravenous LE >1 g/kg/d in adults may result in hepatic steatosis, which is considered as the first step of liver injury (Javid et al., 2005; Kumar and Teckman, 2015; Beath and Kelly, 2016). Compared to LEs being part of the total nutrient admixture, LEs separately taken are prone to medication errors (MEs), especially when the prescription is not under the supervision of a nutrition support pharmacist (Berlana et al., 2019). To our knowledge, separate use is, however, the most common way of LE prescriptions in China (58.01% of patients received PN in a cross-sectional survey among eight tertiary hospitals during 2011–2014) (The Pharmacy Workgroup of CSPEN, unpublished data), which adds to the risk of liver injury. Besides, owing to excessive polyunsaturated fatty acid and linoleic acid content that might increase lipid peroxidation and inflammatory response, soybean oil-based LEs (S-LEs), though providing enough energy and essential fatty acids, have a tendency to cause cellular damage and liver injury (Carter et al., 2007; Wanten and Calder, 2007; Beath and Kelly, 2016). Current evidences from murine models suggest that new generations of LEs, including alternative-LEs (such as medium chain triglycerides-soybean oil LE (MCT-LEs) and olive-soybean oil LE (O-LE)) and fish oil-based LEs (FO-LEs), may improve biochemical measures of hepatobiliary function (Le et al., 2012; Baker et al., 2019). However, data and evidence are still limited especially from routine clinical practice and high quality clinical studies (Javid et al., 2005; Kumar and Teckman, 2015; Kapoor et al., 2019a; b). Therefore, it is important to study the relationship between LEs and liver injury in a real-world setting. To the best of our knowledge, no large population-based studies have been conducted in adults to quantify the risk of hepatic dysfunction following the initiation of LEs.

Prescription sequence symmetry analysis (PSSA) is a valid method used for rapid signal detection of adverse drug events (ADE) by calculating the sequence ratio between exposure and outcomes (Hallas, 1996; Tsiropoulos et al., 2009), which inherently controls time-constant confounders. For now, only two Chinese studies reported drug-related liver injury using PSSA (Fang et al., 2016; Zhang et al., 2019). Moreover, the health insurance data is an important source used for safety evaluation (Tyree et al., 2006). This study aimed to estimate the safety signal between LEs and hepatic protectors using PSSA in a Chinese health insurance database.

## Materials and Methods

### Data Source

This study was conducted by analyzing data from the 2015 Chinese Health Insurance Research Association (CHIRA) database, which is a national-level claims database collecting sampled hospital record of patients from the Urban Employee Basic Medical Insurance scheme all over mainland China (Yang et al., 2018). The data was annually resampled with a two-stage sampling design which has been described previously (Xia et al., 2015; Yong et al., 2018). The 2015 CHIRA database contained a total of 4.64 million patients selected from four municipalities, all provincial capitals, one prefecture-level city of each province, and two county-level cities from each province. The sample proportion was 2%, 5%, and 10%, respectively, of patients from municipalities and provincial capitals, prefecture-level cities, and county-level cities. The database maintains detailed hospital record information of both inpatients and outpatients, including demographics, clinical diagnoses, and prescriptions for drugs and procedures. This study was approved by the Ethical Review Board of Peking University Health Science Center, and informed consent of participant was exempted (IRB00001052-15045). The database was de-identified for protection of patients’ privacy.

### Study Design

We performed an observational study using PSSA to explore the association between LEs and liver injury. PSSA, first proposed by Hallas (1996), is frequently used as a post-marketing active surveillance tool to detect drug safety signals from large prescription databases, with verified validity (Wahab et al., 2013; Pratt et al., 2014). The principles of PSSA have been fully discussed previously (Lai et al., 2017). In brief, it examines the sequence of a marker drug (used to treat an ADE) initiated before and after an index drug (suspected of inducing an ADE): in the absence of a causal association, the propensity to initiate the index drug before or after the marker drug will theoretically be equal in the patient population, showing a symmetrical pattern; however, if the index drug causes the ADE requiring the marker drug for treatment, there will be an asymmetrical prescribing pattern in which more patients will be observed of initiating the index drug before the marker drug. Since PSSA is based on a self-controlled case-only new user design, it is unaffected by potential between-subject confounding and is robust toward time-invariant confounders such as race, gender, and genetic characteristics (Lai et al., 2014).

### Cohort Selection

The index drugs in our study are LEs, consisting of S-LEs, alternative-Les, and FO-LEs (Table 1). The marker drugs are a group of hepatic protectors widely used in China with high specificity as the surrogate of liver injury, chosen by literature search, guideline recommendation, and clinical experience (expert consultation) (Fang et al., 2016; Yu et al., 2017; Zhang et al., 2019), including anti-inflammatory agents, antioxidant agents, antidote agent, choleretics, cell membrane repair agents, and others (Table 1).

**TABLE 1:** Lipid emulsions and hepatoprotective drugs in the study.

Type/class	Drugs in 2015 CHIRA database
Lipid emulsions (LEs)	
Soybean oil-based LEs	Fat emulsion injection (C14–24)
	Fat emulsion and amino acids (18) injection
	Fat emulsion, amino acids, and glucose injection
Alternative-LEs	Medium and long chain fat emulsion injection
	Medium and long chain fat emulsion injection (C8–24)
	Medium and long chain fat emulsion injection (C8–24 Ve)
	Medium and long chain fat emulsion injection (C6–24)
	Structural fat emulsion injection (C6–24)
	Long chain fat emulsion injection (OO)
Fish oil-based LEs	ω-3 fish oil fat emulsion injection
	Multi-oil emulsion injection (C6–24)
Hepatoprotective drugs	
Anti-inflammatory agents	Magnesium isoglycyrrhizinate
	Diammonium glycyrrhizinate
Antioxidant agents	Bicyclol
	Silybin
Antidote agents	Reduced glutathione
Choleretics	Ademetionine
	Ursodeoxycholic acid
Cell membrane repair agents	Polyene phosphatidylcholine
Other agents	Bifendate

LEs, lipid emulsions; CHIRA, Chinese Health Insurance Research Association.

**FIGURE 1:**
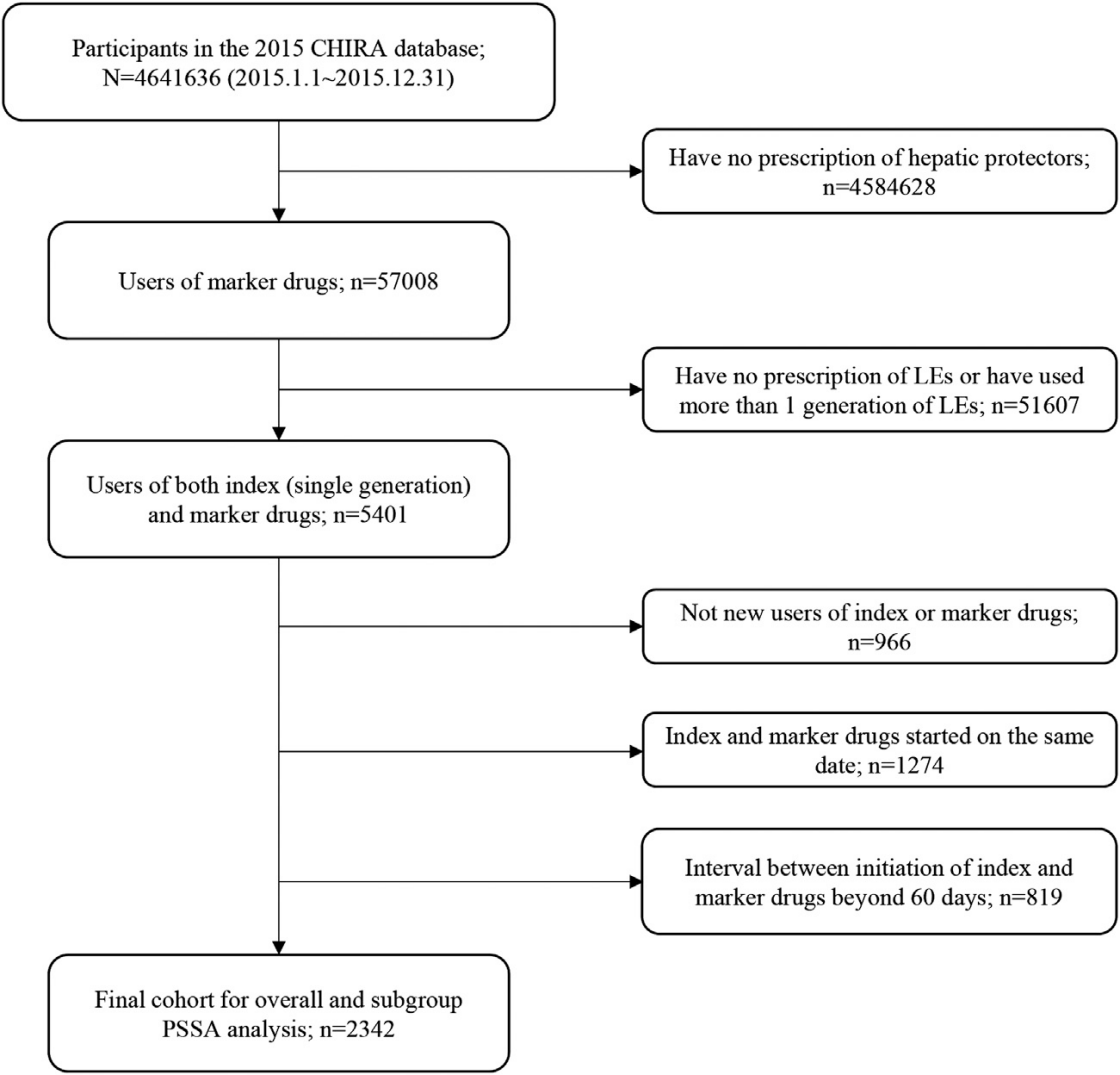
Flowchart of study cohort selection. CHIRA, Chinese Health Insurance Research Association; LEs, lipid emulsions; PSSA, prescription sequence symmetry analysis.

Patients prescribing both an index drug and a marker drug between Jan 1st, 2015, to Dec 31st, 2015, were identified from the 2015 CHIRA database. We applied a washout period based on the “waiting-time distribution” to ensure that patients were new users of the index and marker drugs (Hallas et al., 1997). The observation periods between the initiation of index drugs and marker drugs, namely, time windows, were restricted to 7, 14, 28, 42, and 60 days, respectively, for the sensitivity analysis in consideration of biologic processes leading to steatosis, steatohepatitis, cholestasis, and fibrosis being very likely to occur within 60 days after initiating LEs (Gabe, 2013; Beath and Kelly, 2016) and to reduce within-subject time-variant confounding (Lai et al., 2014). Patients with first prescriptions of the index drug and the marker drug on the same date were excluded because we were not able to determine which drug was prescribed earlier from the data. This helped to lessen patient misclassification induced by prophylactic hepatic protectors. Patients who used more than one generation of LEs (switchers) were also excluded to avoid potential bias from the existing use of the medicine class (Lai et al., 2017). The whole patient selection process and criteria are detailed in Figure 1. For patients included in the final cohort, we extracted their patient unique identification, age, gender, drug prescriptions (generic names with Anatomical Therapeutic Chemical classification codes), and time of prescription.

### Statistical Analysis

The parameter to measure the association between the index drug and the suspected AE in PSSA is called sequence ratio. A crude sequence ratio (CSR) can be calculated by dividing the number of patients initiating LEs first (causal group) by that of those initiating hepatic protectors first (non-causal group). Drug prescribing trends over time can extraneously affect the treatment sequence, and it may result in a biased effect estimate. To adjust for this, we further calculated adjusted sequence ratio (ASR) using a correction method proposed by Tsiropoulos et al., (2009). First, a null-effect sequence ratio (NESR), which represents the expected sequence ratio given no causal association between the index drug and the marker drug, is derived from the overall average probability (*p*) that the index drug will be prescribed before the marker drug in the background population, where *p* is calculated as

$$P=\frac{\sum_{m=1}^{u}\left[LE_{m}\times \left(\sum_{n=m+1}^{m+d}H_{n}\right)\right]}{\sum_{m=1}^{u}\left[LE_{m}\times \left(\sum_{n=m-d}^{m-1}H_{n}+\sum_{n=m+1}^{m+d}H_{n}\right)\right]}.$$

Here, *m* and *n* indicate the consecutive days of the study period, *u* is the last day of the study period, *d* indicates the specified observation time window, *LE*_*m*_ indicates the number of patients receiving first LE prescription on date *m*, and *H*_*n*_ is the number of patients initiating hepatic protectors on date *n*. Given *p*, NESR can be generated as *p*/(1 − *P*). ASR can be calculated as CSR/NESR. The estimation of confidence interval (CI) is based on binomial distribution, using the Wilson (Score) method (Morris and Gardner, 1988; Newcombe, 1998), calculated as

$$\left(\hat{p}+z_{\alpha /2}^{2}/2n\pm z_{\alpha /2}\sqrt{\left(\hat{p}\left(1-\hat{p}\right)+z_{\alpha /2}^{2}/4n\right)/n}\right)/\left(1+z_{\alpha /2}^{2}/n\right)\;$$

Here, *n* is the sample size included in the final analysis and $\hat{p}$ is the ratio of causal group patient number over *n*. Detailed computational formulas have been clearly described in previous studies (Tsiropoulos et al., 2009; Adimadhyam et al., 2019).

Subgroup analyses were conducted by LE generation (S-LEs, alternative-LEs, and FO-LEs), age (≥60 and <60 years), and gender. We performed sensitivity analyses in different time windows to test the robustness of PSSA results and find out during which time period the adverse effects were more likely to occur. ASRs with the lower limit of 95% confidence interval (CI) bigger than 1 were considered statistically significant. All analyses were performed using SAS (version 9.4, SAS Institute Inc., Cary, NC, USA).

## Results

**FIGURE 2:**
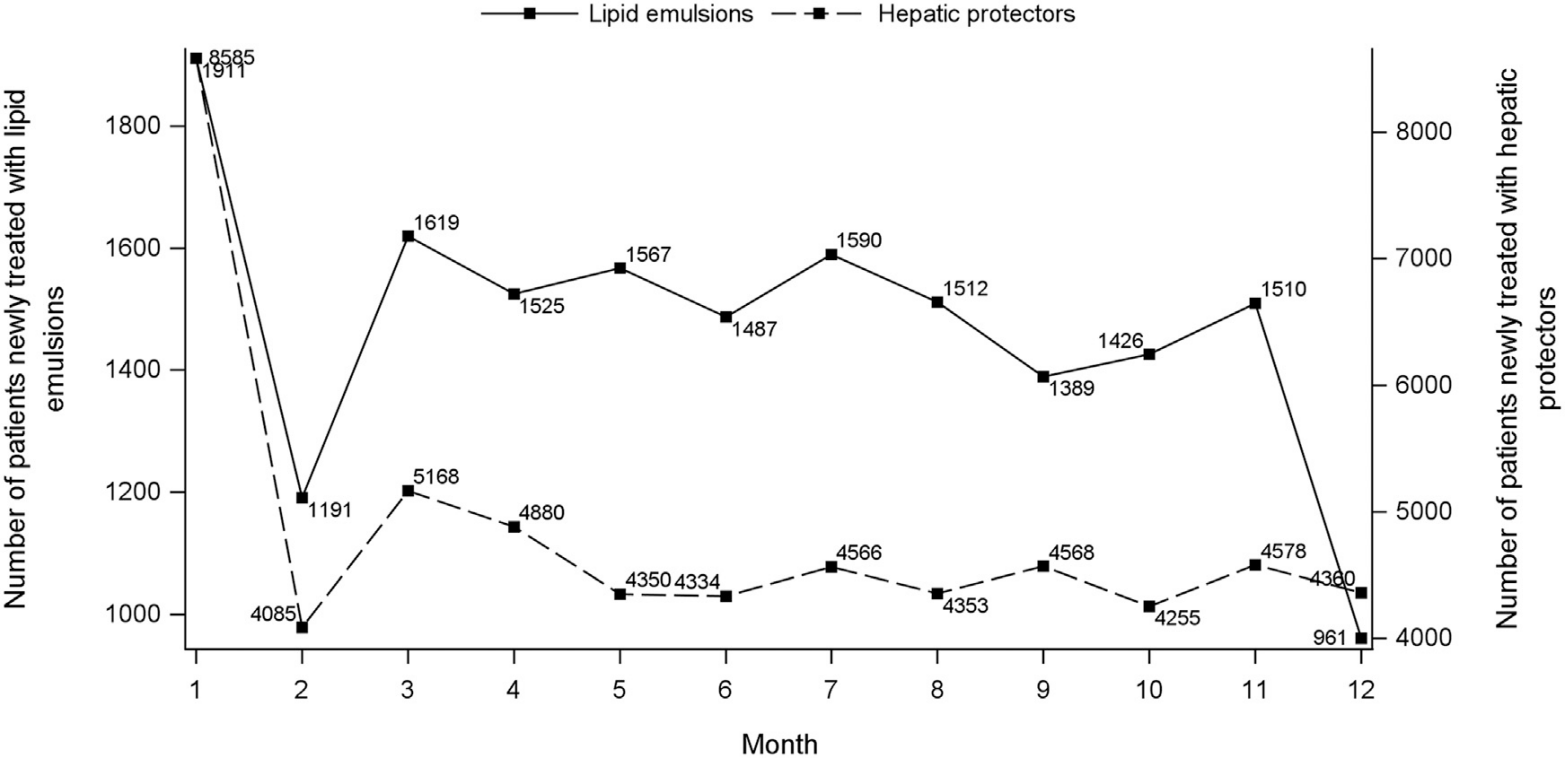
Waiting-time distributions of lipid emulsions and hepatic protectors. For each consecutive month during January to December 2015, the graph depicts the number of persons who presented their first recorded prescription of index (left *Y*-axis) and marker (right *Y*-axis) drug, respectively, during that month.

Figure 1 shows the flowchart of study cohort selection. From the 4.64 million participants covered by the 2015 CHIRA database, we identified 2,342 patients who initiated both a LE and a hepatic protector and met the selection criteria. The waiting-time distributions of both index and marker drugs prescribed in the background population showed a rapid decrease in the first month and reached an almost stable plateau thereafter (Figure 2). Hence, we could infer that many of the patients who filled their initial prescriptions during the first month were prevalence users and should be excluded from our study. Accordingly, the washout period used to exclude prevalent users was set to 1 month. Based on the prespecified time windows, the eligible patients were further grouped into those who started both drugs within 7 (*n* = 416), 14 (*n* = 997), 28 (*n* = 1,697), and 42 (*n* = 2072) days. Of the 2,342 patients with a maximum time window of 60 days, 35.1% were female. The average age was 62.6 ± 15.0 (standard deviation) years. Alternative-LEs were the most commonly used LE in these patients (62.7%), followed by S-LEs (36.5%). FO-LEs were seldom used (0.73%).

**FIGURE 3:**
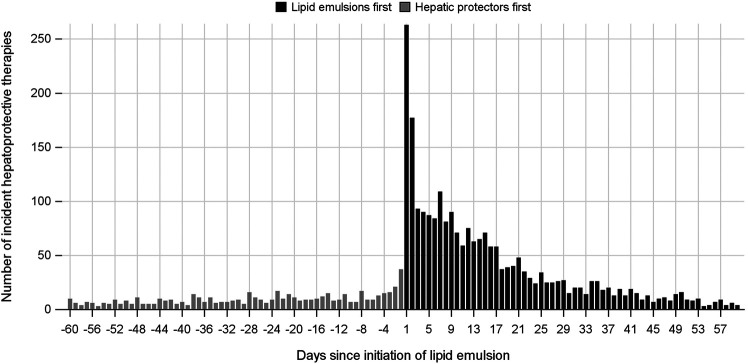
Incident use of hepatic protectors before and after initiation of lipid emulsion within a 60-day time window.

**FIGURE 4:**
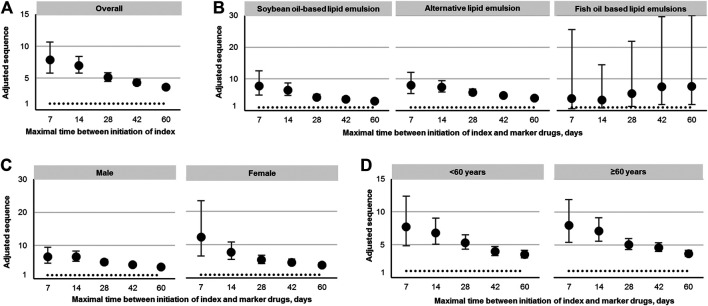
Adjusted sequence ratios. **(A)** Overall, **(B)** by type, **(C)** by gender, and **(D)** by age.

In the analysis where index and marker drugs were initiated within 60 days, the adjusted prescribing trends resulted in an ASR of 3.60 (95% CI 3.26–3.97), indicating that initiating a LE is associated with a 3.6-fold increase in the rate of liver injury receiving hepatic protectors during a 60-day period after initiating LEs. Moreover, a strong asymmetrical pattern of treatment sequence was revealed in Figure 3, showing much more incident hepatoprotective therapies in the months following LEs initiation than before initiation of LEs. An increased risk for hepatic dysfunction following intravenous infusion with LEs was also observed in other prespecified time windows, which was the highest during the first week (ASR, 7.87; 95% CI, 5.80–10.68), and fell with time (Table 2, Figure 4).

**TABLE 2:** Crude and adjusted sequence ratios, overall and by subgroups.

Groups and time intervals	New users of LEs and hepatic protectors	LEs first	Hepatic protectors first	CSR (95% CI)	ASR (95% CI)
Overall					
±7 days	416	370	46	8.04 (5.93–10.91)	7.87 (5.80–10.68)
±14 days	997	874	123	7.11 (5.89–8.58)	6.97 (5.77–8.42)
±28 days	1,697	1,423	274	5.19 (4.56–5.91)	5.12 (4.50–5.83)
±42 days	2072	1,688	384	4.40 (3.93–4.91)	4.32 (3.87–4.83)
±60 days	2,342	1840	502	3.67 (3.32–4.05)	3.60 (3.26–3.97)
Type (generation)					
*S-LEs (1* ^*st*^ *)*					
±7 days	171	152	19	8.00 (4.99–12.83)	7.83 (4.88–12.56)
±14 days	364	316	48	6.58 (4.87–8.91)	6.44 (4.76–8.72)
±28 days	597	485	112	4.33 (3.53–5.32)	4.25 (3.46–5.21)
±42 days	752	594	158	3.76 (3.16–4.48)	3.66 (3.07–4.36)
±60 days	856	650	206	3.16 (2.70–3.69)	3.06 (2.61–3.58)
*Alternative-LEs (2* ^*nd*^ *)*					
±7 days	240	214	26	8.23 (5.49–12.33)	8.05 (5.38–12.07)
±14 days	624	551	73	7.55 (5.92–9.63)	7.42 (5.82–9.47)
±28 days	1,087	927	160	5.79 (4.90–6.85)	5.74 (4.86–6.79)
±42 days	1,303	1,079	224	4.82 (4.17–5.56)	4.78 (4.14–5.52)
±60 days	1,469	1,175	294	4.00 (3.52–4.54)	3.96 (3.49–4.50)
*FO-LEs (3* ^*rd*^ *)*					
±7 days	5	4	1	4.00 (0.60–26.61)	3.85 (0.58–25.60)
±14 days	9	7	2	3.50 (0.83–14.82)	3.42 (0.81–14.47)
±28 days	13	11	2	5.50 (1.37–22.12)	5.44 (1.35–21.89)
±42 days	17	15	2	7.50 (1.91–29.41)	7.54 (1.92–29.56)
±60 days	17	15	2	7.50 (1.91–29.41)	7.65 (1.95–29.99)
Gender					
*Male*					
±7 days	280	244	36	6.78 (4.78–9.60)	6.64 (4.69–9.40)
±14 days	649	565	84	6.73 (5.35–8.45)	6.60 (5.25–8.29)
±28 days	1,110	926	184	5.03 (4.30–5.89)	4.96 (4.23–5.81)
±42 days	1,348	1,089	259	4.20 (3.67–4.81)	4.13 (3.61–4.73)
±60 days	1,520	1,185	335	3.54 (3.13–3.99)	3.46 (3.07–3.91)
*Female*					
±7 days	136	126	10	12.60 (6.69–23.74)	12.32 (6.54–23.21)
±14 days	348	309	39	7.92 (5.69–11.04)	7.77 (5.58–10.83)
±28 days	587	497	90	5.52 (4.41–6.91)	5.45 (4.36–6.82)
±42 days	724	599	125	4.79 (3.95–5.81)	4.72 (3.90–5.73)
±60 days	822	655	167	3.92 (3.31–4.65)	3.86 (3.26–4.57)
Age					
*<60 years*					
±7 days	168	149	19	7.84 (4.89–12.59)	7.71 (4.80–12.37)
±14 days	416	363	53	6.85 (5.14–9.13)	6.77 (5.08–9.02)
±28 days	668	562	106	5.30 (4.31–6.52)	5.27 (4.29–6.49)
±42 days	817	653	164	3.98 (3.36–4.72)	3.96 (3.34–4.70)
±60 days	905	705	200	3.53 (3.01–4.12)	3.51 (3.00–4.11)
≥60 years					
±7 days	248	221	27	8.19 (5.50–12.17)	7.99 (5.37–11.88)
±14 days	581	511	70	7.30 (5.69–9.37)	7.13 (5.56–9.15)
±28 days	1,029	861	168	5.13 (4.34–6.05)	5.02 (4.26–5.93)
±42 days	1,255	1,035	220	4.70 (4.07–5.44)	4.59 (3.97–5.31)
±60 days	1,437	1,135	302	3.76 (3.31–4.27)	3.65 (3.22–4.15)

ASR, adjusted sequence ratio; CI, confidence interval; CSR, crude sequence ratio; LEs, lipid emulsions; FO-LEs, fish oil-based LEs.

In subgroup analyses by the type of LEs initiated (Table 2, Figure 4), all the three generations of LEs showed significantly increased risk of inducing liver injury requiring use of hepatic protectors in all time periods, except for FO-LEs during the first 14 days (ASR, 3.42; 95% CI, 0.81–14.47). ASRs of alternative-LEs were slightly higher than that of S-LEs across all prespecified time windows, and the risks of these two generations decreased over time. ASRs of FO-LEs were much lower than that of S-LEs and alternative-LEs in the first 14 days, but the risk started to rise in longer time windows.

The associations of LEs and hepatic dysfunction were also significant in different age and gender groups, but ASRs differed slightly between groups (Table 2, Figure 4). Generally, ASRs were higher in women and elder patients than men and younger patients across all analysis time windows.

## Discussion

In this large, population-based observational study, we found an association between the index drugs (LEs) and the marker drugs (hepatic protectors), with positive and robust ASRs across all time windows and different generations of LEs, suggesting that LEs might possibly induce hepatic dysfunction in Chinese patients. There was a 3.6- to 7.9-fold increase in the risk of hepatotoxicity within the 2 months after initiating LEs. In 1971, Peden et al. was the first to report a case of an infant who had received total PN for 2.5 months before dying from liver failure (Peden et al., 1971). In a prospective cohort conducted in 90 adults on home parenteral nutrition, 58 patients (65%) developed chronic cholestasis after 6 months of follow-up (Cavicchi et al., 2000). Evidences from different studies also reported that one or more factors relating to hepatic dysfunction, including yet not limited to PN (Lal et al., 2018). Generally, increased intestinal permeability combined with administration of PN promotes lipopolysaccharide and toll-like receptor 4 signaling dependent Kupffer cell activation, which is an early event in the pathogenesis of PN-related liver injury (El Kasmi et al., 2012).

Drug-induced liver injury accounts for approximately 20% of inpatients with acute liver injury in China, owing to a wide use of traditional Chinese medicine and anti-tuberculosis drugs (Li et al., 2007; Yu et al., 2017; Fang et al., 2016; Zhang et al., 2019). The marker drugs used in both studies, and also in our study, were recommended by the Chinese Medical Association guidelines for the treatment of DILI (Yu et al., 2017), and they were widely used to treat liver injury in clinical practice (Fang et al., 2016; Zhang et al., 2019). However, as a signal detection method, PSSA is vulnerable to bias and confounding existing in real-world data (Davies et al., 2019). On one hand, a priori knowledge of the drug-event association may affect the validity of PSSA results, mainly in two ways: 1) the high ASRs observed in our study might be overestimated because prescribers with the knowledge of the ADE might avoid prescribing LEs when the patient already had liver injury; 2) when prescribers with the knowledge of the ADE encounter LE users with liver injury, they might also respond by discontinuing LEs rather than prescribing a liver protection agent, which, on the contrary, would attenuate the signal in our study. On the other hand, fat overload syndrome, which could be caused by rapid infusion (Hojsak and Kolacek, 2014) or high dose (Levit et al., 2016) of LEs, has one clinical manifestation of jaundice and thus might be mistaken as liver injury. This could also lead to an overestimated ASR.

Similar increased risks for hepatic dysfunction showed up in the first 2 months following all the three generations of LEs. However, it is worth noting that a lower risk for hepatic dysfunction was observed in the first 2 weeks following FO-LEs administration, and the absence or reduction of plant sterols in FO-LEs might account for it. Being an important constituent in S-LEs and alternative-LEs, stigmasterol had a prominent role in promoting cholestasis, liver injury, and liver macrophage activation. It might be mediated through suppressing canalicular bile transporter expression (*Abcd11*/BSEP, *Abcd2*/MRP2) via antagonism of the nuclear receptors *(Fxr, Lxr*) and failure of upregulation of the hepatic sterol exporters (*Abcg5/g8*/ABCG5/8)(El Kasmi et al., 2013). Studies in murine models also showed a benefit for liver diseases from FO-LEs (Le et al., 2012; El Kasmi et al., 2013; Baker et al., 2019). As a small sample size in the FO-LEs subgroup, this result was unstable and showed an increased trend after the first 2 weeks. This was in line with the findings of two meta-analyses focusing on the infants, suggesting that FO-LEs, compared with SO-LEs and alternative-LEs, might have an uncertain role in liver complications (Kapoor et al., 2019a; b). A randomized, controlled clinical trial also showed a comparable influence on hepatic function following different generations of LEs in adults with chronic intestinal failure during 1-year follow-up (Hojsak and Kolacek, 2014).

Being opposite to the increasing time trend of risk for liver injury in FO-LEs, the ASRs were highest in the first 14 days after S-LEs or alternative-LEs initiation and decreased thereafter with longer time windows. This suggested that the majority of patients suffered from acute hepatotoxicity after S-LEs or alternative-LEs initiation, possibly resulting from exposure to intravenous LE >1 g/kg/d caused by inappropriate prescription or MEs (rapid infusion speed) (Beath and Kelly, 2016; Hojsak and Kolacek, 2014; Levit et al., 2016). Some patients might discontinue their LEs after first 14-day prescription and were consequently no longer at risk thereafter, also leading to the decreasing trend of ASRs. For most patients, continuous use of LEs might not be necessary, but for some patients, especially those with short bowel syndrome, chronic intestinal pseudo-obstruction, and radiation enteritis, LEs are essential nutrition agents for life support (Matarese et al., 2005; Pironi et al., 2016). Though the increasing trend of ASRs in FO-LEs was in line with the risk from long-term PN reported previously (Javid et al., 2005; Kumar and Teckman, 2015; Beath and Kelly, 2016), the small sample size in the LE subgroup limited its interpretation.

Moreover, the magnitude of risk for liver injury following LEs initiation was higher among female patients and those with elder age. We noted an increased risk of liver injury in the female group, which was also found in a retrospective study conducted in a New York hospital (Huard et al., 2018). The multivariate analysis revealed that female gender was a significant predictor of advanced liver fibrosis among patients receiving PN, who required intestinal transplantation (Huard et al., 2018). Furthermore, altered expression of hepatic β-adrenergic receptors in relation to age-related lipid metabolic dysfunction in liver may explain a slightly higher risk of hepatotoxicity induced by LEs in patients aged more than 60 years (Shi et al., 2018).

Our study has several strengths. To our knowledge, it is the first to evaluate the association of LEs with the potential risk of hepatic dysfunction in a large Chinese population. It provides information on LE-relevant liver injury in a real-world setting that could help us better understand the current situation of safety problems in clinical practice of PN. PSSA has been proved an effective and fast signal detection method in drug safety evaluation, with moderate sensitivity and high specificity (Maclure et al., 2012; Wahab et al., 2013; Pratt et al., 2015). In the rofecoxib and rosiglitazone case, sequence symmetry analysis detected ADE signals earlier than disproportionality analysis (Izyan et al., 2014). As a simple form of a self-controlled design, PSSA is insensitive to between-subject and time-constant confounders (Kubota, 2016). To minimize residual confounding, we also calculated NESR to adjust for the time trends of prescribing. A validation study showed the consistency of PSSA results for detecting ADE regardless of different patterns of medicine utilization and different health settings (Pratt et al., 2015). We further applied a sensitivity analysis with different time windows to test the robustness of the results. Additionally, only drugs used specifically to treat liver injury were selected as marker drugs in our study, to minimize the potential of outcome misclassification.

Our study also has some limitations. Firstly, we only included one-year patient data because the maximum follow-up duration of CHIRA database was one year as a result of the annually resampling data collection strategy. This limited the sample size of study population, especially in the subgroup of FO-LEs, whose effect estimates would thus be more easily affected by random fluctuation. It should also be noted that some delayed events, such as cholestasis, can occur years after PN initiation in adults (Gabe, 2013). However, limiting the study period has the advantage of minimizing the potential of introducing time-dependent confounding factors in PSSA studies (Lai et al., 2017). Moreover, as most hepatic dysfunctions are generally acute events (4–8 weeks) (Palova et al., 2008; Badia-Tahull et al., 2015), one-year follow-up would still be enough to capture related events of interest. Secondly, although a comprehensive set of marker drugs were chosen as a surrogate for hepatotoxicity, we still could not ascertain that it was hepatic dysfunction that provoked the use of these marker drugs in all participants. A cross-validation was not possible because our data did not hold indication of adverse drug reactions, but it would be a valuable topic in future studies. Thirdly, our claims data could not measure over-the-counter use of marker drugs, though most patients indeed got their prescriptions during the hospital stay. Fourth, though the study covers a representative sample from all over China, results should still be generalized with caution as all patient data is from the 2015 CHIRA database, which might be limited to reflect the current prescribing patterns. Fifth, we were not able to explore the dose-response relationship between LEs and hepatotoxicity because of incomplete dosage information.

## Conclusion

Our results show that there is a strong association between LEs and hepatotoxicity with an asymmetrically distributed treatment sequence. The findings suggest that hepatic dysfunction after LEs is common in China, and it is important to strengthen the appropriate use of LEs and enhance patient education at the initiation of LEs to reduce the liver injury.

## Data Availability

The data analyzed in this study is subject to the following licenses/restrictions: The dataset presented in this article is not readily available because of confidentiality agreement between Chinese Health Insurance Research Association database and Peking University. Requests to access these datasets should be directed to http://www.chira.org.cn. The code supporting the conclusions of this article will be made available by the authors. Please send a request to the principal investigator of this article. The steering committee of this study will discuss all requests and decide on the basis of the scientific rigor of the proposal whether code sharing is appropriate. All applicants are asked to sign a code access agreement.

## Abbreviations

ADE, adverse drug event; ASR, adjusted sequence ratio; ATC, anatomical therapeutic chemical; B-LE, borage oil lipid emulsion; CHIRA, Chinese Health Insurance Research Association; CI, confidence intervals; CSR, crude sequence ratio; FO-LE, fish oil-based lipid emulsion; LE, lipid emulsion; MCT-LE, medium chain triglycerides-soybean oil lipid emulsion; ME, medication error; NESR, null-effect sequence ratio; O-LE, olive-soybean oil lipid emulsion; PN, parenteral nutrition; PSSA, prescription sequence symmetry analysis; S-LE, soybean oil-based lipid emulsion.
